# A Pancreatic Tumour Presenting as a Ruptured Spleen

**DOI:** 10.1155/1989/82783

**Published:** 1989

**Authors:** S. Chung, K. Park, A. K. C. Li

**Affiliations:** Department of Surgery The Chinese University of Hong Kong Prince of Wales Hospital Shatin Hong Kong

## Abstract

We report a case of spontaneous rupture of the spleen due to invasion from a pancreatic tumour. We are unaware of any similar case having been reported in the literature.


*HPB Surgery* 1989, Vol. 1, pp. 161–163
Reprints available directly from the publisher
Photocopying permitted by license only

© 1989 Harwood Academic Publishers GmbH
Printed in Great Britain

### CASE REPORT

### A PANCREATIC TUMOUR PRESENTING AS A RUPTURED SPLEEN

S. CHUNG*,[a] K. PARK,[a] and A.K.C. LI*[b]
[a]Lecturer in Surgery; [b]Professor of Surgery

*Department of Surgery, The Chinese University of Hong Kong*
*Prince of Wales Hospital, Shatin, Hong Kong*

*(Received 25 April 1988)*

We report a case of spontaneous rupture of the spleen due to invasion from a pancreatic tumour. We are unaware of any similar case having been reported in the literature.

KEY WORDS: Ruptured spleen, neoplasms, pancreatic.

### INTRODUCTION

Spontaneous rupture of the spleen is an unusual, but well recognised, clinical entity. In most cases it occurs as a result of a pathological process which initially leads to enlargement of the spleen, ie. malaria or infectious mononucleosis. We present a case with a unique aetiology in which the clinical picture was misleading and the diagnosis was only made at laparotomy.

A 53-year-old man was admitted to the medical unit of a near-by hospital for investigation of a pyrexia of 72 hours duration. On physical examination he had a left subcostal mass. Ultrasonography and C.T. scanning showed an irregular mass with loculation which resembled a left subphrenic abscess (Figure 1), despite no preceeding history of surgery or predisposing intrabdominal sepsis. The patient began bleeding from a gastric ulcer with signs of continued blood loss and shock which necessitated transfer to the Department of Surgery, The Prince of Wales Hospital, Hong Kong, where a laparotomy was performed.

At surgery, a 1 cm acute lesser curve gastric ulcer was identified. This was slowly oozing blood and was plicated. In addition there was a large perisplenic haematoma corresponding to the mass identified clinically and on imaging. The spleen had been fragmented by invasion of the hilum from a carcinoma of the pancreatic tail. This blood loss was undoubtedly the cause of the patient's signs of hypovolaemia. A splenectomy and distal pancreatectomy were performed. Histological examination of the specimen confirmed invasion of the spleen by a pancreatic carcinoma.

Post operatively the patient made a good recovery and was discharged on day 10.

* Address correspondence to: Professor A.K.C. Li, Department of Surgery, Prince of Wales Hospital, Shatin, Hong Kong.

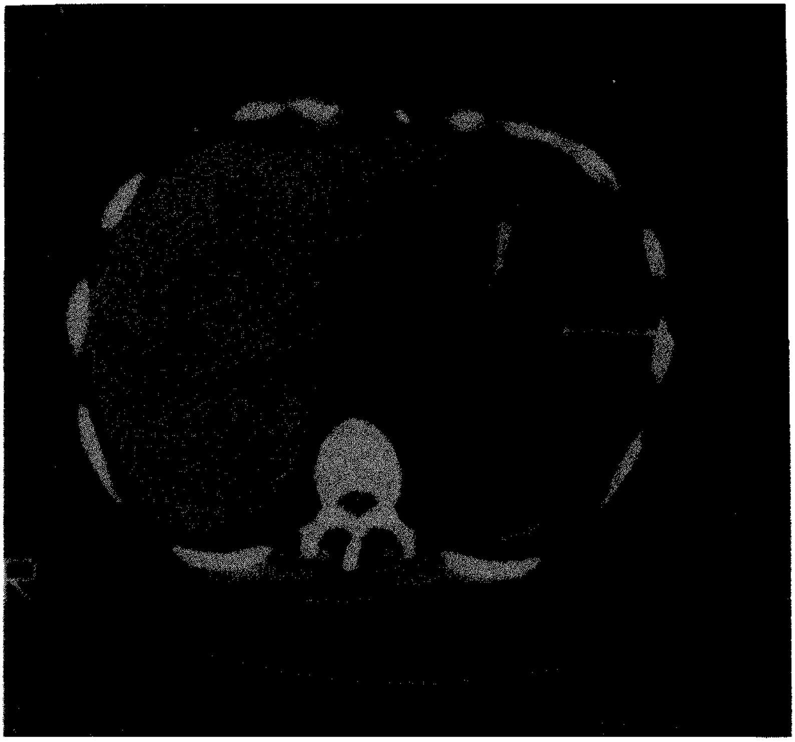

**Figure 1** C.T. Scan Showing Apparent Left Subphrenic Abscess

Unfortunately he was readmitted three months later with abdominal pain and back ache. Further investigations confirmed the clinical diagnosis of recurrent and progressive disease. The patient died two weeks later.

### DISCUSSION

This case serves to emphasize the difficulty in distinguishing between a haematoma and an abscess on C.T. and ultrasound scanning. It also illustrates a very unusual case of splenic rupture due to local invasion from a pancreatic tumour. Spontaneous splenic rupture in association with malignancy classically occurs in leukaemias, but has also been reported in patients with metastatic depositis in the spleen from primary tumours of the lung, bladder, liver and skin.[1,2,3]. The pathological processes involved in these cases have been summarised as: neoplastic invasion of the splenic capsule, splenic infarction, or coagulation disorders.[4] In our patient the spleen was ruptured due to direct invasion of the hilum by a pancreatic neoplasm. Despite the close anatomical relationship of the pancreas to the spleen we are unaware of any previous similar report.

### *References*

1. Karakousis, C.P., Elius, E.G. (1974) Spontaneous rupture of the spleen in malignancies. *Surgery* **76**: 674–7.
2. Rydell, W.B., Ellis, E.G. (1978) Spontaneous rupture of the spleen from metastatic carcinoma. *JAMA* **240**: 53–4.
3. Morie, Y., Snou, T., Hirayama C., Nagasako, R. (1982) Spontaneous rupture of the spleen secondary to metastatic hepatocellular carcinoma: A report of a case and review of the literature. *Am. J. Gastroenterol.* **77**: 882–884.
4. Hynes, R.M., Silver-Stein, N.M., Fawcett, J.F. (1964) Spontaneous Rupture of the Spleen in Leukaemia. *Cancer* **17**: 1356–60.

*Accepted by S. Bengmark on 6 June 1988.*

